# Identification of granulocytic myeloid-derived suppressor cells (G-MDSCs) in the peripheral blood of Hodgkin and non-Hodgkin lymphoma patients

**DOI:** 10.18632/oncotarget.8507

**Published:** 2016-03-30

**Authors:** Olivia Marini, Cecilia Spina, Elda Mimiola, Adriana Cassaro, Giovanni Malerba, Giuseppe Todeschini, Omar Perbellini, Maria Scupoli, Giuseppe Carli, Davide Facchinelli, Marco Cassatella, Patrizia Scapini, Cristina Tecchio

**Affiliations:** ^1^ Department of Medicine, Section of Hematology and Bone Marrow Transplant Unit, University of Verona, Verona, Italy; ^2^ Department of Medicine, Section of General Pathology, University of Verona, Verona, Italy; ^3^ Department of Neurological, Biomedical and Movement Sciences, Section of Biology and Genetics, University of Verona, Verona, Italy; ^4^ Department of Inderdepartmental Laboratory for Medical Research (LURM), University of Verona, Verona, Italy

**Keywords:** granulocytic myeloid-derived suppressor cells, Hodgkin lymphoma, non-Hodgkin lymphoma, low-density neutrophils, normal-density neutrophils

## Abstract

Human granulocytic myeloid-derived suppressor cells (G-MDSCs) have been described as low-density immunosuppressive CD66b^+^CD33^dim^HLA-DR-granulocytes that co-purify with mononuclear cells after density gradient centrifugation of blood from cancer patients. The role of G-MDSCs in Hodgkin (HL) and non-Hodgkin lymphoma (NHL) remains unclear.

The percentage and immunophenotype of CD66b^+^CD33^dim^HLA-DR-cells were analyzed in PBMCs from HL and B-cell NHL patients (*n* = 124) and healthy donors (*n* = 48). The immunosuppressive functions of these cells were tested *in vitro*. Correlations between CD66b^+^CD33^dim^HLA-DR-cells and patient clinicopathological features and outcome, were evaluated.

CD66b^+^CD33^dim^HLA-DR-cells were increased in PBMCs from HL and B-cell NHL patients as compared to healthy donors: 2.18 (0.02–70.92) vs 0.42 (0.04–2.97), *p* < 0.0001. Their percentage remained significantly higher even considering HL (*n* = 31), indolent (*n* = 31) and aggressive (*n* = 62) B-cell NHL patients separately: 1.54 (0.28–26.34), 2.15 (0.02–20.08), and 2.96 (0.25–70.92), respectively, *p* < 0.0001. CD66b^+^CD33^dim^HLA-DR-cells in patient PBMCs were mostly composed of mature CD11b^+^CD16^+^ low-density neutrophils in an activated status, as revealed by their higher CD11b and CD66b expression as compared to conventionally isolated (normal-density) autologous or healthy donor neutrophils. The *in vitro* depletion of CD66b^+^ cells from patient PBMCs restored the proliferation of autologous T cells. Higher frequencies of CD66b^+^CD33^dim^HLA-DR^−^ G-MDSCs correlated significantly with unfavorable prognostic index scores and a shorter freedom from disease progression.

PBMCs from HL and B-cell NHL patients contain a population of CD66b^+^CD33^dim^HLA-DR^−^ G-MDSCs, mostly composed of activated low-density neutrophils with immunosuppressive properties. These findings disclose a previously unknown G-MDSC-mediated mechanism of immune-escape in lymphomas, therefore anticipating possible targets for therapeutic interventions.

## INTRODUCTION

Tumor-induced T cell tolerance is well established in patients with cancer and also in murine tumor models [1]. Increasing evidence suggests that one of the main mechanisms responsible for T cell tolerance towards tumors is the microenvironment-mediated conversion of myeloid cells into potent immunosuppressive cells, known as myeloid derived suppressor cells (MDSCs) [2]. These cells have been recently shown to play a role not only in tumor tolerance - by impairing T cell responses - but also in tumor progression and metastasis [3]. Although authors in the field have defined human MDSCs as immature myeloid lineage-negative (LIN^−^), HLA-DR^−^ and CD11b^+^ or CD33^+^ cells, with an additional division into CD14^low^ monocytic (M-MDSCs) and CD15^+^ granulocytic (G-MDSCs) subsets [3], several types of immunosuppressive myeloid cells characterized by heterogeneous maturation levels and phenotypes, have been documented over the past years in patients with tumors of different origin [4].

Human G-MDSCs, are thought to correspond to low-density, immature [5] and mature [6–9] granulocytes, recovered from the mononuclear cell fraction after density gradient centrifugation of peripheral blood and displaying immunosuppressive activities. Interestingly, even normal-density neutrophils (NDNs), conventionally isolated from the polymorphonuclear cell fraction of peripheral blood, have been found to be T cell suppressive in certain tumors [10–13]. Nonetheless, it is still unclear whether G-MDSCs originate from an altered granulopoiesis or they represent specialized neutrophil subsets [14–17]. In this context, it has been recently proposed to define G-MDSCs based on the lack of HLA-DR antigen, the dim CD33 staining and the expression of CD66b, with CD11b and CD16 being used to differentiate between immature (CD11b^−^ and/or CD16^−^) and mature (CD11b^+^CD16^+^) subsets [16].

While several types of G-MDSCs have been identified in solid tumors [4], G-MDSCs have been poorly investigated in hematological malignancies. To date, G-MDSCs defined as CD11b^+^CD14^−^CD33^+^CD15^+^ [18], CD33^+^CD11b^+^HLA-DR^low/−^CD15^+^ [19], and CD11b^+^CD14^−^HLA-DR^low/−^CD33^+^CD15^+^ [20] cells have been identified in the mononuclear cell fraction of peripheral blood and bone-marrow samples from multiple myeloma patients, while CD11b^+^CD33^+^CD14^−^ G-MDSCs have been isolated from the peripheral blood mononuclear cell fraction [21] and whole peripheral blood [22] of chronic myeloid leukemia patients. Finally, CD11b^+^CD33^+^CD14^−^HLA-DR^−^ G-MDSCs, although not tested for their immunosuppressive activity, have been described in whole blood from Hodgkin lymphoma (HL) patients [23], while functionally-characterized HLA-DR^low^CD11b^+^CD33^+^CD15^+^ G-MDSCs have been recently identified in cryopreserved peripheral blood mononuclear cells (PBMCs) from patients with chronic lymphocytic leukemia [24].

Given the scarce information on the existence of G-MDSCs in lymphomas and on the immunodeficiency status often accompanying such malignancies, herein we examined whether the peripheral blood of patients affected by HL or non-Hodgkin lymphoma (NHL) contains granulocytic-lineage myeloid cells exerting suppressive activities toward T cells. In particular, the aims of our study were: i) to investigate whether PBMCs from patients affected by HL and B-cell NHL contain an increased percentage of CD66b^+^CD33^dim^HLA-DR^−^ cells [16] as compared to healthy donors; ii) to characterize in detail the immunophenotypical features of the CD66b^+^CD33^dim^HLA-DR^−^ cells; iii) to prove their immunosuppressive functions; iv) to evaluate eventual correlations between the percentage of CD66b^+^CD33^dim^HLA-DR^−^ cells and the clinicopathological features, prognostic index scores, disease status, as well as outcome, of patients affected by HL and B-cell NHL.

## RESULTS

### The percentage of CD66b^+^CD33^dim^HLA-DR^−^ cells is increased in PBMCs from lymphoma patients as compared to healthy donors

**Figure 1:**
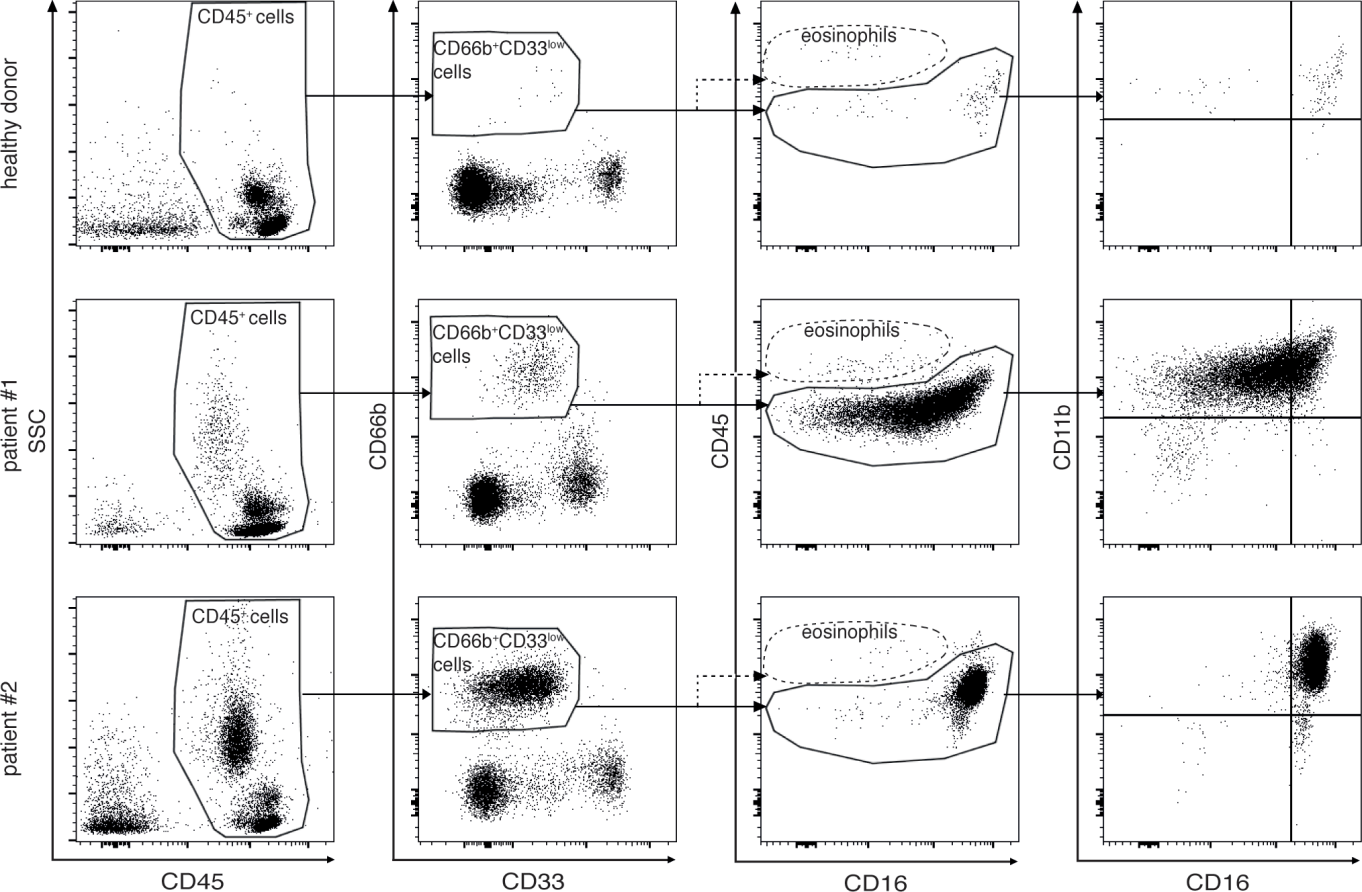
Gating strategies applied to analyze by flow cytometry PBMCs from healthy donors (top panel row) and lymphoma patients (middle and bottom panel row) After exclusion of doublets and dead cells by morphological parameters (left and second to left panel columns), granulocytic cells were identified as CD45^+^CD66b^+^CD33^dim^ cells (second to right panel column). Eosinophils were excluded from the analysis based on their bright CD45 and the dim CD16 expression (second to right panel column). CD66b^+^CD33^dim^ cells were further analyzed with respect to CD16 and CD11b in order to identify different stages of differentiation (right panel column). Middle and bottom panels are representative of samples obtained from patients with a lower and a higher percentage of CD66b^+^CD33^dim^HLA-DR^−^ cells, respectively.

PBMCs of both healthy donors and lymphoma patients at diagnosis were analyzed according to the gating strategies reported in Figure 1 and Supplementary Figure 1, in order to establish whether they could contain candidate G-MDSCs. In brief, within total CD45^+^ PBMCs we gated on CD66b^+^CD33^dim^ cells (Figure 1). Notably, CD66b^+^CD33^dim^ cells were HLA-DR^−^ as compared to autologous CD66b^−^CD33^high^ monocytes (Supplementary Figure 1). Eosinophils were then excluded from the analysis based on their bright CD45 expression [25] (Figure 1).

**Figure 2:**
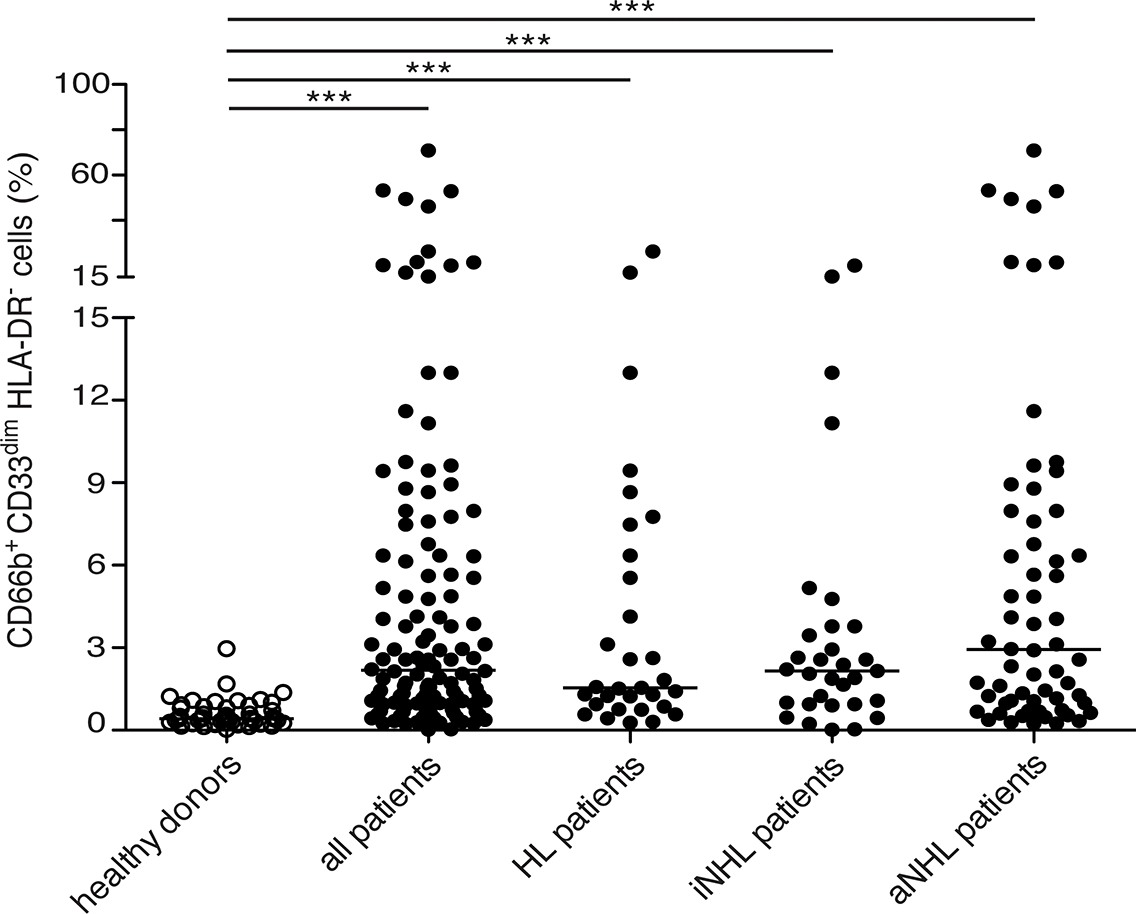
Median percentage of CD66b^+^CD33^dim^HLA-DR^−^ cells with respect to CD45^+^ PBMCs of healthy donors (*n* = 48) as compared to: the whole series (*n* = 124) of lymphoma patients (*p* < 0.001), patients affected by HL (*n* = 31, *p* < 0.001), and indolent (*n* = 31, *p* < 0.001) or aggressive B-cell NHL (*n* = 62, *p* < 0.001), respectively iNHL: indolent B-cell NHL; aNHL: aggressive B-cell NHL.

The percentages of CD66b^+^CD33^dim^HLA-DR^−^ cells within PBMCs from healthy donors (*n* = 48) and the whole cohort of lymphoma patients (*n* = 124) were then compared (Figure 2). According to our analysis, despite the strong variability from patients to patients (second and third panel rows of Figure 1, and Figure 2), overall the median percentage of CD66b^+^CD33^dim^HLA-DR^−^ cells was significantly higher in PBMCs from patients at diagnosis as compared to healthy donors [2.18 (0.02–70.92) *vs* 0.42 (0.04–2.97), *p* < 0.0001]. CD66b^+^CD33^dim^HLA-DR^−^ cells were in fact very poorly represented in healthy donors (Figure 1, top panel row). Interestingly, the difference was significant even when the median percentage of CD66b^+^CD33^dim^HLA-DR^−^ cells within PBMCs of patients affected by HL [1.54 (0.28–26.34), *p* < 0.0001], and either indolent [2.15 (0.02–20.08), *p* < 0.0001] and aggressive B-cell NHL [2.96 (0.25–70.92), *p* < 0.0001] were compared to healthy donors (Figure 2). On the other hand, no correlation was observed between the percentage of CD66b^+^CD33^dim^HLA-DR^−^ cells within PBMCs and the neutrophil or total leukocyte counts (*p* = 0.138 and *p* = 0.086, respectively) obtained by the simultaneous analysis of peripheral blood samples from the same patients.

Overall, these findings indicate that PBMCs from patients affected by HL and B-cell NHL contain a population of granulocytic cells displaying a phenotype consistent with that of G-MDSCs.

### CD66b^+^CD33^dim^HLA-DR^−^ cells within PBMCs from lymphoma patients represent a heterogeneous population of granulocytic cells in different stages of maturation, with a significant prevalence of the mature component

CD66b^+^CD33^dim^HLA-DR^−^ cells within PBMCs from lymphoma patients at diagnosis displayed a wide variability with respect to the side scatter parameter (SSC) by flow cytometric analysis (Figure 1, left panel column), as well as morphological features consistent with a population of granulocytic cells in different stages of maturation (Supplementary Figure 2). Therefore, we performed an evaluation of CD11b and CD16 expression (Figure 1, right panel column) in order to discriminate among different maturation stages, with CD11b^−^CD16^−^, CD11b^+^CD16^−^ and CD11b^+^CD16^+^ representing the immature, intermediate, and mature subpopulation, respectively [16]. Notably, given that the gating strategies used allowed excluding eosinophils from our analysis (Figure 1), we will henceforward describe CD11b^+^CD16^+^ cells within CD66b^+^CD33^dim^HLA-DR^−^ cells as mature low-density neutrophils (LDNs).

**Figure 3:**
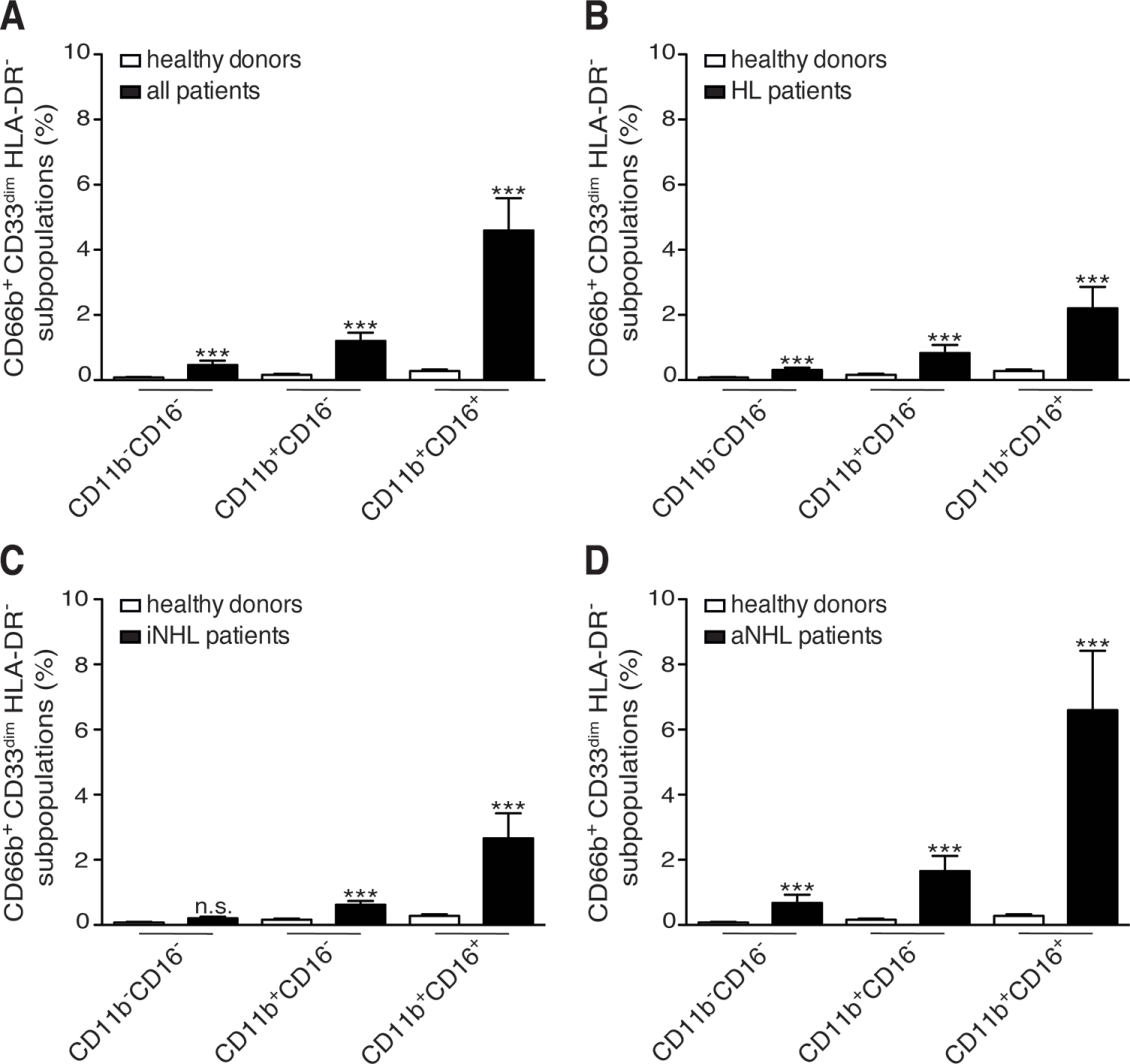
Mean percentages with SEM of CD66b^+^CD33^dim^HLA-DR^−^ cells subpopulation (CD11b^−^CD16^−^, CD11b^+^CD16^−^, and CD11b^+^CD16^+^) in PBMCs (**A**) healthy donors (*n* = 48) and total patients (*n* = 124); (**B**) healthy donors (*n* = 48) and HL patients (*n* = 31); (**C**) healthy donors (*n* = 48) and patients (*n* = 31) with indolent B-cell NHL (iNHL); (**D**) healthy donors (*n* = 48) and patients (*n* = 62) with aggressive B-cell NHL (aNHL).

In our cohort of lymphoma patients, CD66b^+^CD33^dim^HLA-DR^−^ cells within PBMCs were mostly composed of CD11b^+^CD16^+^ LDNs (54.80 ± 2.97%, mean ± SD percentage), at significantly higher levels than the CD11b^+^CD16^−^ (30.74 ± 2.30%) and CD11b^−^CD16^−^ (13.19 ± 1.54%) subpopulations, (*p* < 0.0001). Remarkably, CD11b^+^CD16^+^ LDNs resulted the most represented subpopulation of CD66b^+^CD33^dim^HLA-DR^−^ cells even considering patients affected by HL, indolent and aggressive B-cell NHL lymphomas separately (data not shown). Interestingly, CD11b^+^CD16^+^ LDNs appeared particularly enriched in PBMCs from patients with a higher percentage of CD66b^+^CD33^dim^HLA-DR^−^ cells (Figure 1, bottom panel row). The percentages of CD66b^+^CD33^dim^HLA-DR^−^ subpopulations with respect to PBMCs were therefore compared in healthy donors and patients (Figure 1, top panel row and Figure 2). As expected the mean percentage of each subpopulation was significantly higher in the whole series of lymphoma patients as compared to healthy donors: CD11b^+^CD16^+^ (4.59 ± 0.99 *vs* 0.28 ± 0.04, *p* < 0.0001), CD11b^+^CD16^−^ (1.21 ± 0.25 *vs* 0.16 ± 0.03, *p* < 0.001), and CD11b^−^CD16^−^ (0.47 ± 0.13 *vs* 0.08 ± 0.01, *p* = 0.004) (Figure 3A). Similar results were obtained by analyzing the different lymphoma types (i.e. HL and B-cell NHL, either indolent and aggressive) separately (Figure 3B–3D). Overall, these data suggest that, in lymphoma patients, the expansion of CD66b^+^CD33^dim^HLA-DR^−^ cells within PBMCs is sustained by the proportional increment of the 3 subpopulations, with CD11b^+^CD16^+^ LDNs being the most represented.

### The *in vitro* depletion of CD66b^+^ cells from PBMCs of lymphoma patients restores the proliferation of autologous T lymphocytes, therefore identifying a population of G-MDSCs

**Figure 4:**
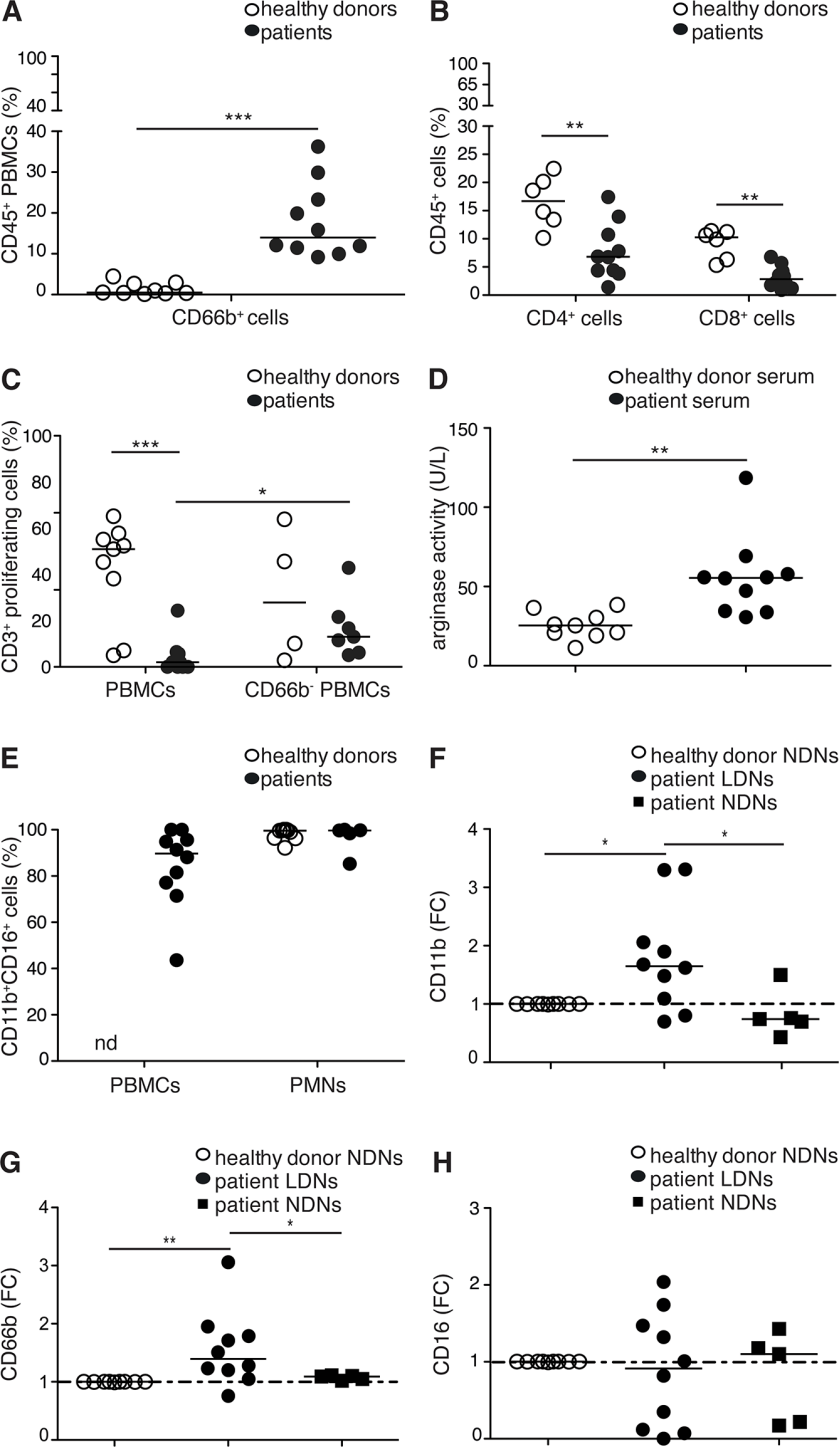
(**A**, **B**) Median percentage of CD66b^+^ cells within CD45^+^ PBMCs (A) or CD4^+^ and CD8^+^ cells within total CD45^+^ cells (B) of healthy donors (*n* = 9 and 6, respectively) and lymphoma patients (*n* = 10). (**C**) Median percentage of proliferating CD3^+^ T cells in CD3/CD28-stimulated PBMCs (total or CD66b^+^ cell depleted) from healthy donors (*n* = 9 and 4, respectively) and lymphoma patients (*n* = 10 and 7, respectively), as revealed by CFSE dilution measured by flow cytometry. (**D**) Median arginase activity measured in serum from healthy donors (*n* = 9) and lymphoma patients (*n* = 10). (**E**) Median percentage of CD11b^+^CD16^+^ LDNs with respect to CD66b^+^CD33^dim^HLA-DR^−^ cells in the mononuclear fraction (PBMCs), and median percentage of CD11b^+^CD16^+^ NDNs in the polymorphonuclear fraction (PMNs) of healthy donors (*n* = 9 and 9, respectively) and lymphoma patients (*n* = 10 and *n* = 5, respectively). (**F**–**H**) Expression of CD11b, CD66b and CD16 on NDNs from healthy donors (*n* = 9) and LDNs or NDNs from lymphoma patients (*n* = 10 and 5, respectively). Data are expressed as median fold change (FC) of the median fluorescence intensity (MFI) of each antigen on patient LDNs or NDNs over NDNs from healthy donors. * = *p* ≤ 0.05; ** = *p* ≤ 0.01; *** = *p* ≤ 0.001. nd: not done.

We subsequently investigated whether CD66b^+^CD33^dim^HLA-DR^−^ cells within PBMCs of lymphoma patients manifested immunosuppressive properties. For this purpose, we selected a small cohort of HL and B-cell NHL patients at diagnosis containing a high percentage of CD66b^+^ cells in the PBMC fraction (≥ 5%) (Figure 4A). Interestingly, the percentage of CD4^+^ and CD8^+^ T cells and their ability to proliferate upon stimulation with anti-CD3/CD28 antibodies, were reduced in these patients as compared to healthy donors (Figure 4B, 4C). In addition, an elevated arginase activity was detected in serum samples simultaneously obtained from the same patients (Figure 4D). Importantly, CD66b^+^ cell depletion from lymphoma patient PBMCs significantly enhanced T cell proliferation (Figure 4C). By contrast, CD66b^+^ cell depletion from PBMCs of patients containing a low percentage of the latter cells (< 5%), or from PBMCs of healthy donors, did not affect T cell proliferation (Figure 4C and data not shown). A detailed immunophenotypical characterization of the immunosuppressive CD66b^+^CD33^dim^HLA-DR^−^ cells revealed that they were mostly composed of CD11b^+^CD16^+^ LDNs similar to autologous or healthy donor NDNs (Figure 4E). To better characterize patient LDNs with respect to their immunophenotype and activation status, we then analyzed the levels of expression of CD11b, CD66b, and CD16 on patient LDNs and autologous or healthy donor CD11b^+^CD16^+^ NDNs [5, 6], according to the gating strategies shown in Supplementary Figure 3. Consistently with their activated status [26], patient CD11b^+^CD16^+^ LDNs displayed significantly higher levels of CD11b and CD66b as compared to autologous and healthy donor CD11b^+^CD16^+^ NDNs, while no changes in CD16 expression were observed (Figure 4F–4H and Supplementary Figure 3). Overall, these data suggest that PBMCs from lymphoma patients at diagnosis include a population of CD66b^+^CD33^dim^HLA-DR^−^ cells, mostly composed of mature activated CD11b^+^CD16^+^ LDNs, which can be defined as G-MDSCs given their suppressive properties on T cell proliferation.

### The percentage of CD66b^+^CD33^dim^HLA-DR^−^ cells in PBMCs from lymphoma patients at diagnosis correlates with prognostic index scores, freedom from disease progression (FFDP) and disease status

**Figure 5:**
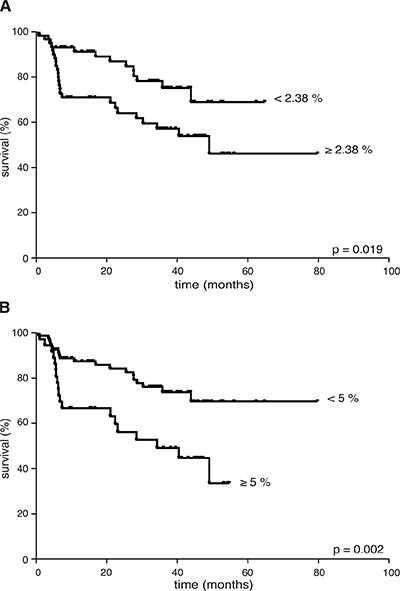
FFDP in 117 lymphoma patients evaluable for disease outcome according to: (**A**) the median percentage of CD66b^+^CD33^+^HLA-DR^−^ cells (i.e. 2.38% of PBMCs), or (**B**) the minimum effective percentage of CD66b^+^CD33^+^HLA-DR^−^ cells (i.e. 5% of PBMCs) for *in vitro* T cell inhibition experiments. CD66b^+^CD33^+^HLA-DR^−^ cells percentage was < 2.38% in 59 patients, < 5% in 81 patients.

Considering the whole cohort of lymphoma patients, an increased percentage of CD66b^+^CD33^dim^HLA-DR^−^ cells did not correlate with advanced clinical stages [I–II *vs* III–IV: 1.72 (0.25–70.92) *vs* 2.47 (0.02–46.21), *p* = 0.95], bulky disease [present *vs* no: 2.35 (0.28–49.34) *vs* 2.18 (0.02–70.92), *p* = 0.688], extranodal [present *vs* no: 1.49 (0.25–70.92) *vs* 2.57 (0.02–53.35), *p* = 0.34], splenic [present *vs* no: 2.47 (0.02–46.21) *vs* 2.02 (0.23–70.92), *p* = 0.52], or bone-marrow involvement [present *vs* no: 2.57 (0.02–21.61) *vs* 2.14 (0.25–70.92), *p* = 0.71], and systemic symptoms [present *vs* no: 3.09 (0.25–21.41) *vs* 1.85 (0.02–70.92), *p* = 0.163]. By contrast, a higher CD66b^+^CD33^dim^HLA-DR^−^ cell percentage correlated with unfavorable prognostic index scores [Hasenclaver, FLIPI or IPI ≥ 3 (*n* = 37) *vs* < 3 (*n* = 87): 3.12 (0.25–70.92) *vs* 1.69 (0.02–53.35), *p* = 0.036], thus suggesting a prognostic role for these cells in terms of disease outcome. To verify such a hypothesis, we first analyzed the FFDP of all patients evaluable for outcome (*n* = 117) with respect to the median CD66b^+^CD33^dim^HLA-DR^−^ cell percentage in their PBMCs at diagnosis (i.e. 2.38%). As a result, patients having a CD66b^+^CD33^dim^HLA-DR^−^ cell percentage higher than the median experienced a significantly worse FFDP than the remaining (*p* = 0.019) (Figure 5A). The significance of CD66b^+^CD33^dim^HLA-DR^−^ cells increased (*p* = 0.002) when we used as a cut-off the minimal percentage required for an effective *in vitro* function (i.e. 5%) (Figure 5B). With respect to the latter cut-off, with the limits of the small samples size, significant differences in FFDP were observed even considering HL (*n* = 31) and aggressive B-cell NHL (*n* = 60) patients separately (*p* = 0.038 and *p* = 0.026, respectively), while the difference did not reach the statistical significance (*p* = 0.083) in the evaluable indolent B-cell NHLs (*n* = 26) in which the percentage of G-MDSCs was ≥ 5% only in 5 patients. With disease status regard, we could analyze the percentage of CD66b^+^CD33^dim^HLA-DR^−^ cells in a representative sample of patients (*n* = 34) who achieved a complete remission of disease. According to our analysis the median percentage of CD66b^+^CD33^dim^HLA-DR^−^ cells was significantly lower in PBMCs from patients in complete remission - after at least 3 months since the completion of first-line therapy -, as compared to the same patients at the time of diagnosis [0.65 (0.03–14.99) *vs* 1.79 (0.25–70.92), *p* = 0.0004] (Supplementary Figure 4).

Next, we investigated whether one of the subpopulations composing CD66b^+^CD33^dim^HLA-DR^−^ cells could have a major significance with regard to the same variables we analyzed above. According to our analysis, neither the mean percentage of mature CD11b^+^CD16^+^ LDNs, nor intermediate CD11b^+^CD16^−^ or immature CD11b^−^CD16^−^ cell subsets correlated with any patient clinicopathological characteristics or prognostic scores (data not shown). However, patients with a CD66b^+^CD33^dim^HLA-DR^−^ cell percentage higher than the frequencies used as cut-off for FFDP analysis (i.e. ≥ 2.38 and ≥ 5) showed a significantly higher percentage of CD11b^+^CD16^+^ LDNs than patients with a CD66b^+^CD33^dim^HLA-DR^−^ cell percentage below the same cut-off (64.84 ± 4.45 *vs* 46.01 ± 3.56, *p* = 0.0013 and 71.32 ± 5.29 *vs* 48.58 ± 3.31, *p* = 0.0006, respectively) (Supplementary Table 1). As expected, an inverse distribution was observed with respect to CD11b^+^CD16^−^ and CD11b^−^CD16^−^ subpopulations in patients above and below the same cut-off (Supplementary Table 1).

## DISCUSSION

Novel areas of research for lymphoma patients include improving the efficacy of adoptive cellular therapies, modulating T regulatory cells, developing novel lymphoma vaccine and enhancing tumor-specific innate immune response [27]. In the latter context, this study reports that an expanded population of immunosuppressive CD66b^+^CD33^dim^HLA-DR^−^ cells is present within PBMCs of lymphoma patients, either affected by HL or B-cell NHL. To the best of our knowledge the existence of a population of functionally characterized CD66b^+^CD33^dim^HLA-DR^−^ G-MDSCs was not previously described in lymphomas. By contrast, mature CD14^+^HLA-DR^(low)/−^ monocytes with tested immunosuppressive activity (M-MDSCs) were previously reported in the peripheral blood of 40 patients with aggressive and indolent B-cell NHL [28], therefore allowing to hypothesize the presence of both G-MDSCs and M-MDSCs in such a context, as already observed in solid tumors [4, 29].

We found that CD66b^+^CD33^dim^HLA-DR^−^ cells in PBMCs from lymphoma patients include a population of granulocytic cells in different stages of maturation, mostly composed of mature CD11b^+^CD16^+^ cells (i.e. LDNs). Noteworthy, a further characterization of the latter cells allowed us to ultimately define CD66b^+^CD33^dim^HLA-DR^−^CD11b^+^CD16^+^ cells as activated LDNs, given their higher expression of CD11b and CD66b as compared to autologous and healthy donor-derived NDNs. Interestingly, populations of mature activated granulocytes, endorsed with G-MDSCs characteristics, were previously described in the mononuclear cell fraction of peripheral blood samples from solid tumor patients [6, 9]. According to the aforementioned studies, a status of activation was responsible for the phenotypic and functional changes - consistent with those of G-MDSCs - observed in these populations of low-density granulocytes, which were shown to inhibit T cells via H_2_O_2_ production [8] or arginase release from their intracellular granules [6–7, 9]. In agreement with the latter observations, we also found an elevated arginase activity in sera obtained from a representative sample of HL and B-cell NHL patients, suggesting that activated neutrophil subsets (i.e. LDNs) could exert an arginase-mediated immunosuppressive activity even under lymphoma settings. Obviously, the prevalence of CD11b^+^CD16^+^ LDNs in CD66b^+^CD33^dim^HLA-DR^−^ G-MDSCs raises several questions. For instance, including the immunosuppressive role in tumors of mature LDNs or NDNs and their mutual relationship [15], their kinship [17] with conventionally defined G-MDSCs (e.g., immature myeloid cells expressing granulocytic markers) [3], and finally the need for a definite consensus about their classification as G-MDSCs or specialized/activated neutrophils.

In our study, independently from the lymphoma type (i.e. HL or NHL), we observed a lack of association between the percentage of CD66b^+^CD33^dim^HLA-DR^−^ cells or their subpopulations, and patient clinicopathological characteristics, including clinical stage, systemic symptoms, bone-marrow, spleen or extranodal involvement, and bulky disease. However, a higher percentage of CD66b^+^CD33^dim^HLA-DR^−^ cells was associated with unfavorable prognostic scores and, mostly, with a significantly worse FFDP, therefore suggesting that at least in lymphomas, the generation of G-MDSCs is more related to the biological aggressiveness of the disease than to its size. Accordingly, among aggressive B-cell NHL patients, those affected by a particularly unfavorable type of diffuse large B-cell lymphoma (DLBCL) (i.e. “double” and “triple expressor” DLBCLs, *n* = 7) [30], had a significantly higher percentage of circulating CD66b^+^CD33^dim^HLA-DR^−^ cells as compared to the remaining 47 DLBCL patients [7.59 (2.57–46.21) vs 2.14 (0.25–70.92), *p* = 0.045] (O.M. and C.T. personal communication). Interestingly, in a representative sample of 34 patients of our series, the percentage of CD66b^+^CD33^dim^HLA-DR^−^ cells was significantly lower at the achievement of complete remission as compared to the time of diagnosis, when the disease was active. Unfortunately, the apoptotic effects of chemotherapy on MDSCs [31] and the design of the study did not allow us to analyze peripheral blood samples of patients with disease progression under treatment or relapse. However, the significantly higher percentage of CD66b^+^CD33^dim^HLA-DR^−^ cells that we detected in PBMCs from a new series of 12 relapsed patients as compared to healthy donors [2.75 (0.24–46) *vs* 0.42 (0.04–2.97), *p* < 0.0002] (O.M. and C.T. personal communication), further support the role of lymphoma in sustaining the development of G-MDSCs.

Worthy of note, the correlation that we reported between the percentage of circulating CD66b^+^CD33^dim^HLA-DR^−^ G-MDSCs at the time of diagnosis and disease prognostic scores and outcome, suggests that the latter cells may be a suitable prognostic marker in HL and B-cell NHL. In contrast to our observation, the only report analyzing G-MDSCs in lymphomas found, in a cohort of 60 HL patients, a prognostic significance for immature CD34^+^ MDSCs but not for G-MDSC [23]. However, it should be noted that the latter G-MDSCs were identified by whole blood flow cytometry as CD11b^+^CD33^+^CD14^−^HLA-DR^−^ cells, therefore making it difficult for any comparison with our data. Moreover, we acknowledge the fact that the prognostic significance of either CD66b^+^CD33^dim^HLA-DR^−^ G-MDSCs or whole blood CD11b^+^CD33^+^CD14^−^HLA-DR^−^ G-MDSCs will be definitely established only by future studies evaluating larger series of lymphoma patients.

In our cohort, patients having a worse FFDP included a significantly higher percentage of CD11b^+^CD16^+^ LDNs within their G-MDSCs than patients having a better FFDP. These clinical observations would confirm what suggested by our *in vitro* experiments, in which immunosuppressive CD66b^+^ cells were found to be mostly composed of activated LDNs. Moreover, in the same patients, the expansion of the latter cells was associated with a significantly decreased percentage of the subpopulations at the intermediate and immature stages of maturation, thus suggesting their rapid lymphoma-mediated recruitment to a mature and activated status.

Overall, our results demonstrate that in HL and B-cell NHL patients a population of functionally defined G-MDSCs mainly composed of mature activated LDNs is present. Besides identifying a possible prognostic marker, these findings disclose a previously unknown G-MDSC-mediated mechanism of immune-escape in lymphomas, therefore anticipating future targets for therapeutic interventions. Further studies are awaited in order to establish whether CD66b^+^CD33^dim^HLA-DR^−^ G-MDSCs could be inhibited or reprogrammed for therapeutic purposes.

## MATERIALS AND METHODS

### Patients and healthy donors

Between February 2010 and December 2014, 124 patients referred to our Institution for a new diagnosis of lymphoma, (31 HL, 93 B-cell NHL), were prospectively enrolled to this study upon informed consent. The study received approval from the Ethics Board of the Azienda Ospedaliera Universitaria Integrata di Verona (Project 2371). Criteria of inclusion were: a new diagnosis of HL or B-cell NHL, and age ≥ 16 years. Criteria of exclusion were: the presence of infections, rheumatological disorders, malignancies other than lymphoma, concurrent or previous steroid treatment, and ongoing chemotherapy. Patient characteristics are reported in Table 1. Forty-eight sex- and age-matched healthy volunteer donors were also enrolled at the Blood Bank of our Institution at the time of blood donation, upon informed consent.

**Table 1:** Patient demographics and clinicopathological characteristics

Lymphoma Classification	Clinicopathological Features	Patient number and age
Hodgkin*		31
	Median age (range)	38 years (16–79)
	Male/Female	22/9
	I–II	20
	III–IV	11
	Bulky disease	9
	Bone-Marrow involvement	1
	Spleen involvement	9
	Extranodal involvement	4
	Systemic symptoms	14
Non Hodgkin (B-cell)**		93
	Median age (range)	68 years (23–88)
	Male/Female	60/33
Indolent		31
	I–II	16
	III–IV	24
	Bulky disease	–
	Bone-Marrow involvement	18
	Spleen involvement	14
	Extranodal involvement	6
	Systemic symptoms	3
Aggressive		62
	I–II	27
	III–IV	35
	Bulky disease	25
	Bone-Marrow involvement	9
	Spleen involvement	17
	Extranodal involvement	30
	Systemic symptoms	15
**Total patients**		124
	Median age (range)	64 years (16–88)
	Male/Female	82/42

* Hodgkin lymphoma: classic type (*n* = 25), lymphocyte predominant (*n* = 6).

** non Hodgkin lymphoma indolent: follicular grade 1-3a (*n* = 12), marginal zone (*n* = 17), and mucose-associated lymphoid tissue lymphoma (*n* = 2);

**non Hodgkin lymphoma aggressive: follicular grade 3b (*n* = 8), diffuse large B cell (DLBCL) (*n* = 54, including: 1 MYC and BCL-2 and 3 MYC and BCL-6 double expressors, and 3 MYC, BCL-2 and BCL-6 triple expressors).

Histological diagnoses were done according to WHO criteria [32]. Based on a clinically-oriented classification [33], the 93 B-cell NHL cases were subsequently divided in the indolent [*n* = 31] and aggressive [*n* = 62] subgroups and analyzed accordingly. Among aggressive B-cell NHLs, diffuse large B cell lymphomas (DLBCL) were immunohistochemically defined “double expressors” and “triple expressors” based on their expression of MYC and BCL-2 or BCL-6, and MYC, BCL-2 and BCL-6, respectively [30].

Patients underwent complete blood count and blood chemistry measurements. Clinical staging included physical examination, computed tomography scans of chest, abdomen, pelvis and brain, positron emission tomography, and bone-marrow biopsy. Patients were staged according to the Ann Arbor staging system [34]. The term “bulky” was referred to lymphomas in the chest that were at least one-third as wide as the chest, or lymphoma in other areas that were at least 10 centimeters across.

At diagnosis, the Hasenclever Prognostic Index [35] was calculated for patients affected by HL, while the Follicular Lymphoma International Prognostic Score (FLIPI) [36] and the International Prognostic Score (IPI) [37] were calculated for indolent and aggressive B-cell NHL patients, respectively. Both patients affected by HL and B-cell NHL, were treated according to the European Society Medical Oncology (ESMO) guidelines [38]. Patients who underwent, an intensive high-dose sequential chemotherapy with stem cell autografting [39] as upfront treatment (i.e. patients affected by mantle cell lymphoma) were excluded from the study.

Peripheral blood samples from patients at the time of diagnosis or healthy donors were freshly obtained by venipuncture in EDTA-treated sterile tubes. After at least 3 months since the completion of first-line chemotherapy (and/or radiotherapy whenever delivered) and in case of complete remission achievement, patients were required to undergo a further venipuncture for peripheral blood sample collection.

Serum samples from patients at the time of diagnosis or age- and sex-matched healthy donors were obtained after collection of peripheral blood by venipuncture in gel and clot activator tubes, promptly centrifuged at 1800 g for 10 minutes, and stored at −80°C until used.

### G-MDSCs identification by flow cytometry

For both patients and healthy donors, granulocytes and PBMCs were freshly obtained from 3 ml of whole blood, collected in EDTA and rigorously processed within 2 hours over centrifugation on Ficoll-Paque solution (GE Healthcare Life Sciences, Cleveland, Ohio), according to manufacturer's protocol. G-MDSCs were identified in PBMCs based on the lack of HLA-DR, the dim CD33 staining, and the expression of CD66b, with CD11b and CD16 being used to differentiate between immature (CD11b^−^ and CD16^−^), intermediate (CD11b^+^/CD16^−^) and mature (CD11b^+^CD16^+^) subsets (16). PBMCs (0.5 × 10^6^ cells/tube) were stained for 15 minutes with: CD16 PerCP-Cy5.5 (clone 3G8), CD33 PE-Cy7 (clone P67.6), CD11b APC (clone D12), HLA-DR PE (clone L243), and CD45 APC-H7 (clone 2D1) all from BD Biosciences (Becton, Dickinson and Company, Franklin Lakes, New Jersey) and with CD66b FITC (clone g10f5, BioLegend, San Diego, CA).

Fluorescence acquisition was performed using a FACS Canto flow cytometer (Becton, Dickinson and Company, New Jersey): it was considered reliable when at least 0.25 × 10^6^ CD45^+^ events were recorded. The analysis of flow cytometric data was performed using FlowJo software (Tree Star, Inc. Ashland, OR).

### Morphology

To assess cell morphology, cytospins of PBMCs from patients at diagnosis or healthy donors were obtained by centrifuging 5 × 10^4^ cells on microscope slides and stained by the Giemsa/May-Grunwald procedure. Pictures were taken using a Leica DFC 30FX Digital Color Camera on a Leica DM 6000 B microscope at a 40× magnification and processed with Adobe Photoshop (Adobe Systems).

### MDSCs depletion and T cell proliferation assay

Depletion of MDSCs from freshly isolated PBMCs of patients at diagnosis or healthy donors were performed by labeling PBMCs with anti-CD66b-FITC followed by anti-FITC magnetic bead separation, according to the specifications of the manufacturer (Miltenyi Biotech). The percentage of CD66b^+^ cells in the negative selected population was < 1%.

To measure T cell proliferation, PBMCs (depleted or not of CD66b^+^ cells) freshly isolated from patients at the time of diagnosis or healthy donors were labeled with carboxy-fluorescein diacetate succinimidyl ester (CFSE) (CellTrace™ CFSE Cell Proliferation Kit, Life Technologies) according to manufacturer's instructions. CFSE-labeled PBMCs were stimulated with mAbs anti-CD3 (30 ng/ml) and anti-CD28 (100 ng/ml) (both from Sanquin Blood Supply, Netherlands), for 96 hours. Cells were then collected and stained with anti-CD4 and anti-CD8 mAbs (BD Biosciences, Becton, Dickinson and Company, Franklin Lakes, New Jersey, USA) for 15 minutes at 4°C. After propidium iodide (Life Technologies, Carlsbad, USA) positive cells exclusion, CFSE fluorescence was measured on gated CD4^+^ or CD8^+^ T cells. The percentage of proliferating T cells was calculated as the percentage of CFSE negative cells with respect to total CD4^+^ or CD8^+^ T cells. Fluorescence acquisition (at least 0.25 × 10^6^ events) was performed using a MACSQuant^®^ Flow Cytometer (Miltenyi Biotec, Bergisch Gladbach, Germany). The analysis of flow cytometric data was performed using FlowJo software (Tree Star, Inc. Ashland, OR).

### Arginase activity

Thawed serum samples (100 μl) were loaded on Amicon^®^ Ultra-0.5 Centrifugal Filter Devices (Merck-Millipore, Darmstadt, Germany), diluted to 500 μl with H_2_O and centrifuged at 14,000 g for 30 min to remove urea. Concentrated samples were diluted with H_2_O and tested for arginase activity as previously described (40,41). Arginase activity (Units per Liter of sample, U/L) was defined as the amount of enzyme able to convert 1 μmole of L-arginine to ornithine and urea per minute at pH 9.5 and 37°C.

### Statistics

To perform the association analyses, values not showing a normal distribution were log transformed and the Shapiro-Wilk test was used to test their normality. Differences of quantitative normally distributed variables between 2 or more groups were tested by Student's *t*-test or one-way ANOVA, respectively. When variables resulted to be not normally distributed, even after log transformation, non-parametric rank-based tests were used on untransformed values. In particular for unpaired data, Mann-Whitney and Kruskal-Wallis tests were used to compare 2 or more than 2 groups respectively. Non-parametric Freidman test used when comparing paired data.

Associations between quantitative variables were assessed using regression linear models. Covariates were included into the models when relevant.

The percentage of CD11b^−^CD16^−^, CD11b^+^CD16^−^, and CD11b^+^CD16^+^ subpopulations was expressed as either percentage of CD66b^+^CD33^dim^HLA-DR^−^ cells or percentage of mononuclear CD45^+^ cells according to the association investigated.

Freedom from disease progression (FFDP) was defined as the interval from the initiation of the primary treatment to the first recurrence of disease (progression or relapse) [35]. Response criteria were established according to Cheson et al. [42]. Patients in complete remission were censored on June 30th 2015. Overall 117/124 (94.3%) patients were evaluable for disease outcome at that time. Among the 7 patients not evaluable for clinical outcome, 1 with aggressive B-cell NHL was lost to follow-up, 1 with aggressive B-cell NHL refused chemotherapy due to the old age, and 5 with indolent B-cell NHL were not considered for analysis because they were lost to follow-up (*n* = 2) or because they did not require any treatment (*n* = 3). Survival distributions of two samples were compared using the log-rank Mantel–Cox test. Statistical analyses were performed using the R package version 3.2.0 [43] and GraphPad Prism 5.0 software (GraphPad Software, Inc, California).

## SUPPLEMENTARY MATERIALS FIGURES AND TABLES


