# Supplementary material for: Identification of granulocytic myeloid-derived suppressor cells (G-MDSCs) in the peripheral blood of Hodgkin and non-Hodgkin lymphoma patients
Source: Oncotarget. 2016 Mar 30;7(19):27676–88. doi: 10.18632/oncotarget.8507 (PMC5053680; doi:10.18632/oncotarget.8507)
Supplement: Supplementary file 1 [file oncotarget-07-27676-s001.pdf]

Identification of granulocytic myeloid-derived suppressor cells (G-MDSCs) in the peripheral blood of Hodgkin and non-Hodgkin lymphoma patients

Supplementary Materials

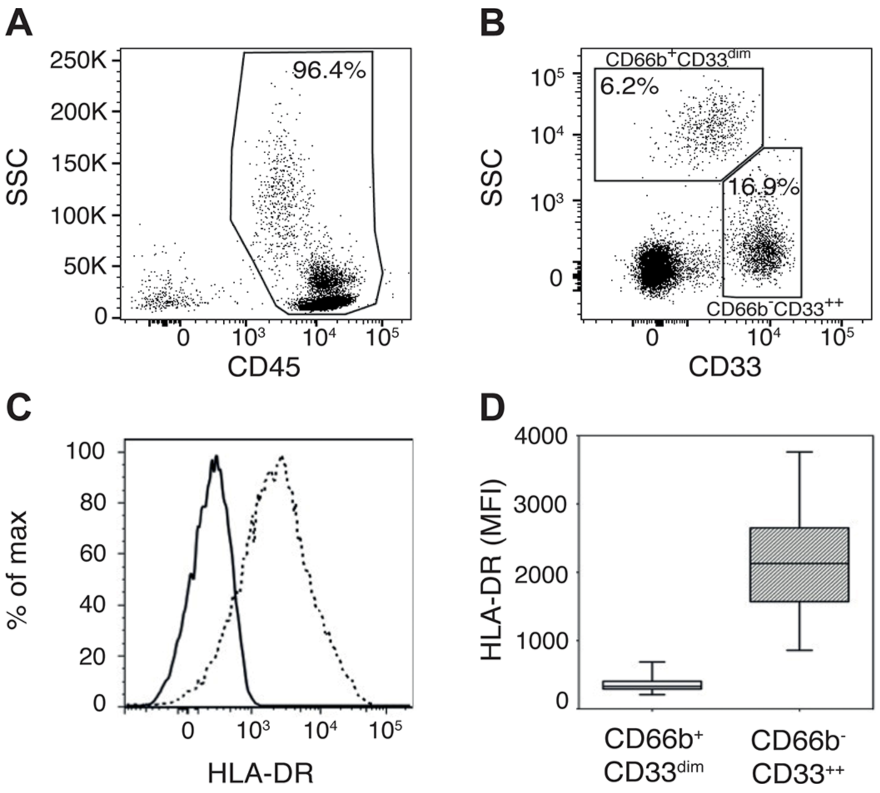

Supplementary Figure S1: Analysis of HLA-DR expression by CD66b^{+}CD33^{dim} cells (low-density granulocytes) as compared to CD66b^{-}CD33^{++} cells (monocytes).

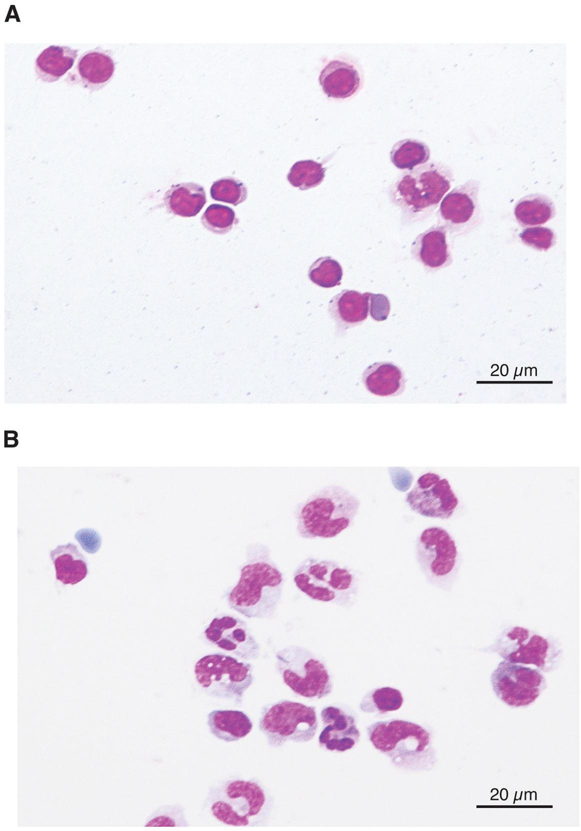

Supplementary Figure S2: Cytospins showing the morphological characteristics of PBMCs obtained from peripheral blood samples of: (A) a representative healthy donor; (B) a representative lymphoma patient. Giemsa/May-Grumwald. Magnification 40×. Scale bar, 20 μm.

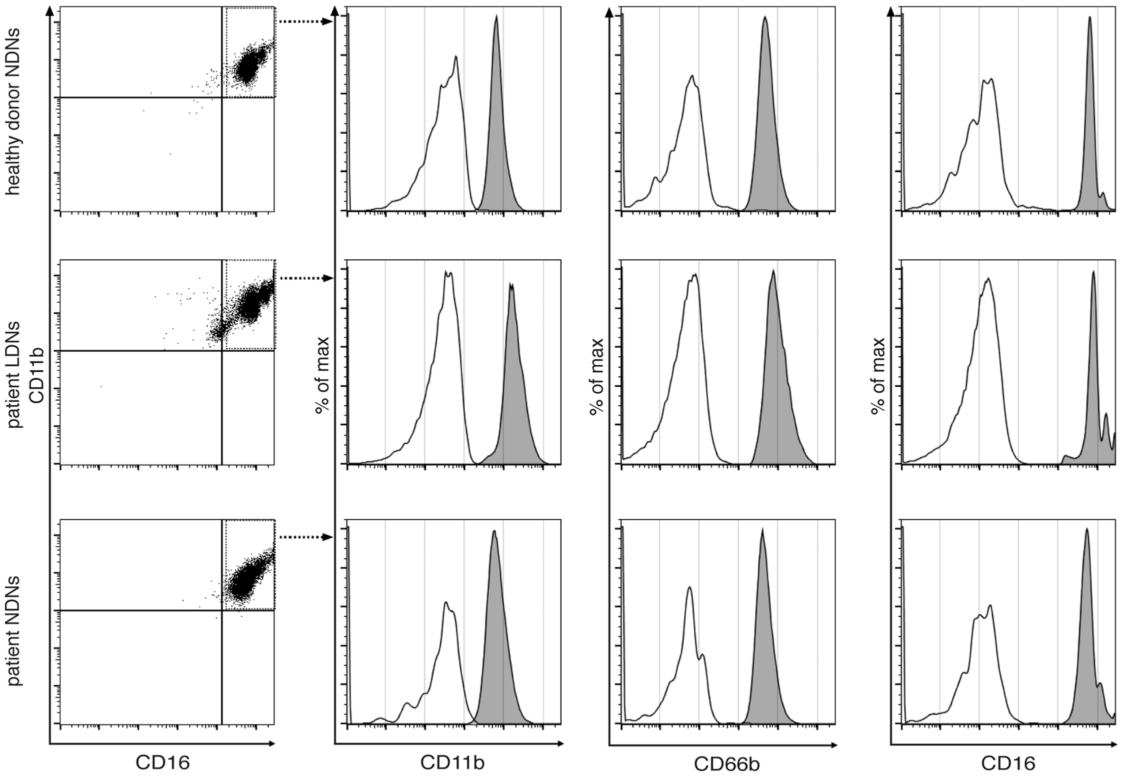

Supplementary Figure S3: Gating strategies applied to analyze by flow cytometry the level of expression of CD11b, CD66b, CD16 (grey histograms), or relative isotype controls (open histograms), by mature CD66b^{+}CD33^{dim}HLA-DR^{-} CD11b^{+}CD16^{+} NDNs from healthy donors (top panel row) or LDNs (middle panel row) and NDNs (bottom panel row) from lymphoma patients.

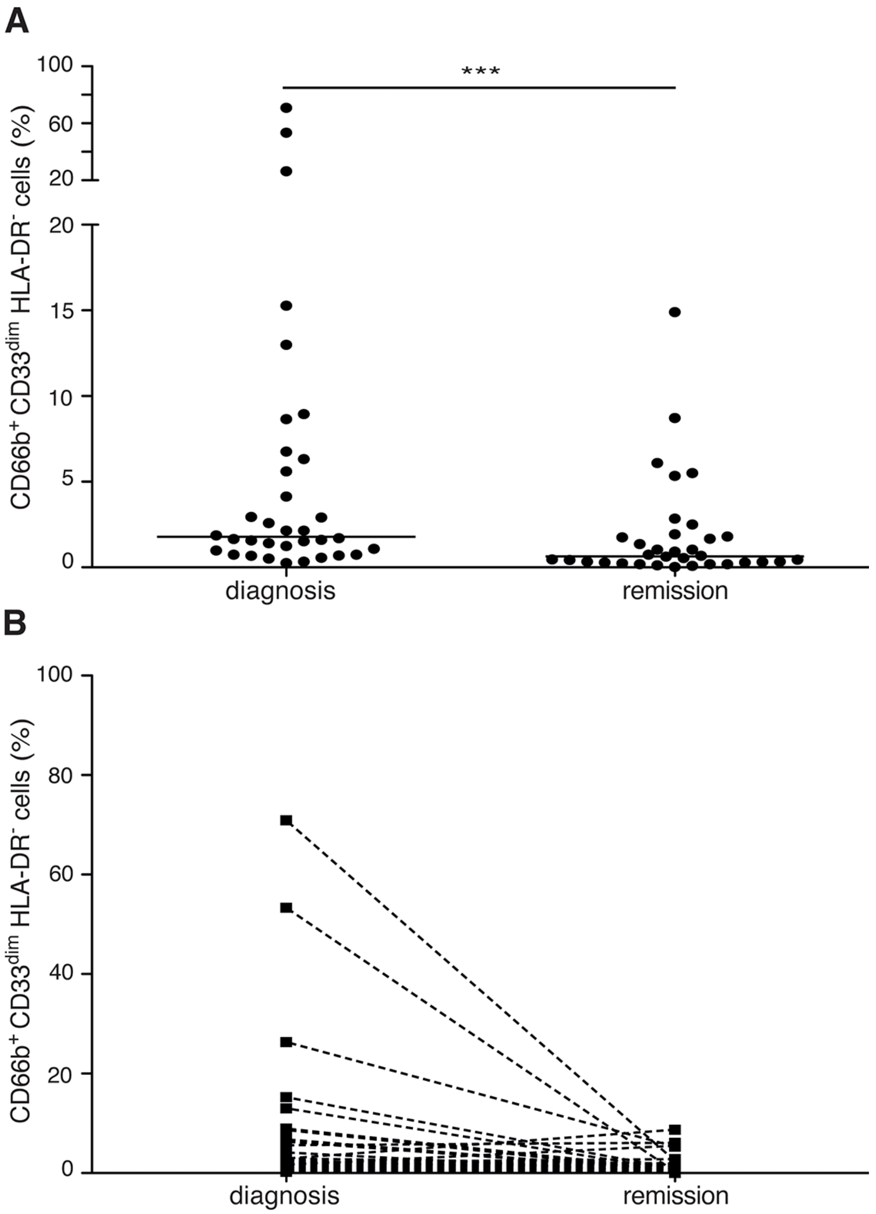

Supplementary Figure S4: (A) Median frequency of CD66b$^+$CD33^{dim}HLA-DR$^-$ cells in PBMCs from lymphoma patients (n = 34: HL 9, indolent NHL 6, aggressive NHL 19) at the time of diagnosis, and at the complete remission achievement. (**B**) Trend in frequency of CD66b$^+$CD33^{dim}HLA-DR$^-$ cells with respect to each of the 34 patients at the time of diagnosis, and at the complete remission achievement. ***$p \leq 0.001$.

Supplementary Table S1: Composition of CD66b$^+$CD33^{dim}HLA-DR$^-$ G-MDSCs in terms of CD11b$^-$CD16$^-$, CD11b$^+$CD16$^-$, and CD11b$^+$CD16$^+$ subpopulations in patients with a median frequency of CD66b$^+$CD33^{dim}HLA-DR$^-$ cells under *vs* equal or above the values used as cut-off for FFDP analysis

CD66b$^+$/CD33^{dim}/HLA-DR$^-$ (cut-off)	CD11b$^-$/CD16$^-$ (mean ± SEM)	CD11b$^+$/CD16$^-$ (mean ± SEM)	CD11b$^+$/CD16$^+$ (mean ± SEM)
≥ 2.38 *vs* < 2.38	9.69 ± 2.27 *vs* 15.35 ± 1.84 p = 0.056	23.60 ± 3.53 *vs* 37.95 ± 2.85 p = 0.0021	64.84 ± 4.45 *vs* 46.01 ± 3.56 p = 0.0013
≥ 5 *vs* < 5	5.71 ± 1.57 *vs* 15.42 ± 1.90 p = 0.002	20.07 ± 4.32 *vs* 35.40 ± 6.67 p = 0.0039	71.32 ± 5.29 *vs* 48.58 ± 3.31 p = 0.0006
